# EglN2 contributes to triple negative breast tumorigenesis by functioning as a substrate for the FBW7 tumor suppressor

**DOI:** 10.18632/oncotarget.14290

**Published:** 2016-12-27

**Authors:** Mamoru Takada, Ming Zhuang, Hiroyuki Inuzuka, Jing Zhang, Giada Zurlo, Jinfang Zhang, Qing Zhang

**Affiliations:** ^1^ Lineberger Comprehensive Cancer Center, University of North Carolina School of Medicine, Chapel Hill, NC 27599, USA; ^2^ Department of General Surgery, Xinhua Hospital, Shanghai Jiaotong University School of Medicine, Shanghai, China; ^3^ Department of Pathology, Beth Israel Deaconess Medical Center and Harvard Medical School, Boston, Massachusetts 02215, USA; ^4^ Department of Pathology and Laboratory Medicine, University of North Carolina, Chapel Hill, NC 27599, USA

**Keywords:** EglN2, triple negative breast cancer, FBW7

## Abstract

EglN2 contributes to ERα-positive breast tumorigenesis by acting as an estrogen-inducible gene. However, the detailed molecular mechanism(s) underlying the post-transcriptional regulation of EglN2 and its potential role in Triple Negative Breast Cancer (TNBC) remains largely unclear. By using C3Tag transgenic mice and tumor-derived C3Tag cell line, here we report that EglN2 contributes to TNBC tumor progression and genetic knockout of *EglN2* improves C3Tag mice survival from tumor progression. Mechanistically, we further show that FBW7, an E3 ligase complex component that is frequently downregulated in TNBC, negatively regulates EglN2 protein stability. As such, depletion of *FBW7* in breast cell lines leads to upregulation of EglN2 and other canonical FBW7 substrates. Conversely, FBW7 overexpression leads to EglN2 downregulation in a GSK3β-dependent manner. Furthermore, we identified some potential serine or threonine residues on the C-terminal of EglN2 that may mediate its binding and potential regulation by FBW7. Together, our study reveals that EglN2 might act as an FBW7 ubiquitin ligase substrate contributing to TNBC.

## INTRODUCTION

Triple Negative Breast Cancer (TNBC), the subtype tested negative for estrogen receptors (ER), progesterone receptors (PR) or Her2, accounts for 15-25% of breast cancers [1, 2]. It is more likely to affect younger people, African Americans and those with a *BRCA1* gene mutation [1, 2]. TNBC is well known for its aggressive clinical behavior and early peak of disease recurrence. Furthermore, TNBC does not respond to endocrine therapy, such as Tamoxifen or aromatase inhibitors, or to therapies that target the HER-2 oncoprotein, such as Herceptin [2]. Due to the lack of optimal therapeutic targets, TNBC represents a specific subtype of breast cancer with worst prognosis [1, 2]. Therefore, there remains an urgent question to be addressed: Can we identity important biological features that serve as high value targets for the development of novel treatment modalities for TNBC? This line of research carries significant social and economic importance.

Hypoxia is one of the major characteristics of solid tumors [3]. Upon sensing the oxygen tension, cancer cells adapt to the stressful environment and proliferate uncontrollably. The key oxygen sensors include three EglN family members in humans (termed as EglN1, EglN2, and EglN3), which are members of iron and 2-oxoglutarate-dependent dioxygenases that catalyze substrate hydroxylation and mediate their stability [4, 5]. For example, hydroxylation of the HIFα (hypoxia-inducible factor α) by EglN1 generates a binding site for a ubiquitin ligase containing the von Hippel Lindau (VHL) protein [4]. Previous research show that EglN2 is an ER-inducible gene [6, 7]. Moreover, our own research demonstrate that EglN2 contributes to ER positive breast tumorigenesis by inducing cyclin D1 transcription [8, 9]. However, the potential post-transcriptional regulation of EglN2 and its physiological role in other subtypes of breast cancer remain largely unknown.

F-box and WD repeat domain-containing 7 (FBW7) is a well-established tumor suppressor in diverse mouse and human cancers [10–15]. There are some cancer types that harbor high rate of *FBW7* mutations/deletions, including T-cell acute lymphoblastic leukemia (T-ALL), colon cancer, endometrial cancer and cholangiocarcinomas [10, 11, 16]. Mechanistically, FBW7 belongs to one of F-box proteins that is an essential component of SCF (SKP1-CUL1-F-box) E3 ligase complex, which target proteins for ubiquitination and degradation [17]. In recent years, there are some FBW7 E3 ligase substrates identified, including Cyclin E1, c-Jun, c-Myc, Notch1 and Mcl-1 [18]. Loss of *FBW7* in cancer leads to aberrant accumulation of these substrates, accounting for the tumor phenotypes observed in xenograft or genetic mouse models [18]. In breast cancer specifically, FBW7 was reported to target mTOR for proteasomal degradation [19]. Interestingly, in human breast cancer cell lines and primary breast tumors, there exists a reciprocal correlation between *FBW7* loss and *PTEN* loss, which also activates mTOR [19]. In addition, the *FBW7* genetic locus is in a region that is frequently deleted in TNBC [20]. This suggests an important role of FBW7 in breast cancer.

In this paper, by using both xenograft and mouse models, we showed that EglN2 contributes to TNBC breast cancer progression. Mechanistically, *FBW7* loss in TNBC cell lines leads to increased EglN2 protein levels.

## RESULTS

### EglN2 contributes to TNBC tumor growth

**Figure 1:**
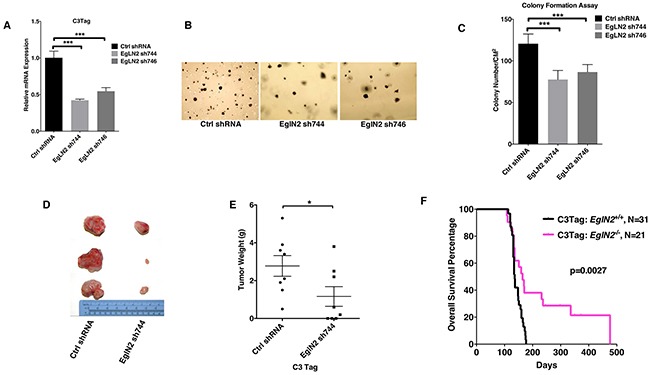
EglN2 contributes to TNBC tumor growth **A, B, C**. qRT-PCR (A), representative images (B) and quantification (C) of soft agar assays from C3Tag cells infected with the lentivirus encoding EglN2 shRNAs (744, 746) or Control (Ctrl) shRNA. The statistical significance was calculated using student's t test. *** denotes p value of < 0.005. Error bars represent one SEM. **D, E**. Representative tumor gross appearance and tumor weight plots for C3Tag cells infected with the lentivirus encoding EglN2 shRNA (744) or Control (Ctrl) shRNA implanted into the mammary fat pads in FVB/NJ mice. * denotes p value of <0.05 for comparison between two groups by using unpaired two sample t-test. Error bars represent one SEM. **F**. Kaplan-Meier survival curves for C3Tag: *EglN2*^+/+^ and C3Tag: *EglN2*^−/−^ mice. The p value was determined by the Log-rank (Mantel-Cox) test.

To examine whether EglN2 could play a functional role in TNBC, we isolated C3 Tag cell lines from mammary tumors of a transgenic line that mimics human TNBC development as described previously [21]. *EglN2* depletion by two independent hairpins (sh744 and sh746) in this cell line significantly decreased anchorage independent growth (Figure 1A-1C). Next, allograft experiments by using *EglN2*-depleted cell line displayed decreased tumor growth and tumor weight compared to the control cell line (Figure 1D-1E). These results support the important role of EglN2 in TNBC tumor progression, which motivated us to cross C3 Tag mice with *EglN2* WT or *EglN2* knockout mice. To this end, we generated a cohort of C3Tag: *EglN2^+/+^* and C3Tag: *EglN2^−/−^* mice. While C3Tag: *EglN2^+/+^* mice displayed the median survival of 136 days, C3Tag: *EglN2^−/−^* mice showed much longer survival rate with the median survival of 169 days (p=0.0027, Log-rank Mantel-Cox test) (Figure 1F). It is worth noting that there appears to be a biphasic tumor development for C3Tag: *EglN2^−/−^* mice. For the short-term survival, there is no distinctive difference between C3Tag: *EglN2^−/−^* with C3Tag: *EglN2^+/+^* mice but overtime, there appears to be a group of mice with prolonged long-term survival with C3Tag: *EglN2^−/−^* mice. One possibility is that EglN2 knockout may not benefit those fast-growing tumors but may offer long-term benefits for other slower-growing tumors. These results suggest the important role of EglN2 regulating TNBC tumor growth.

### EglN2 protein stability is negatively regulated by FBW7

**Figure 2:**
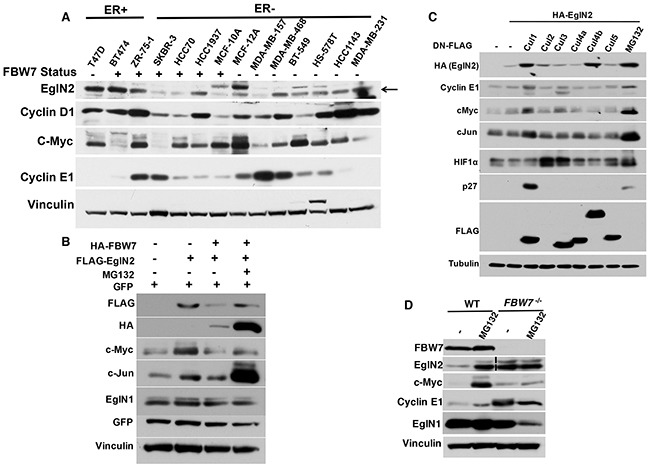
EglN2 Protein Stability is Negatively Regulated by FBW7 **A**. Immunoblots from a panel of indicated breast cancer cell lines. **B**. Immunoblots of cell lysates from 293T cells transfected with FLAG tagged EglN2, HA tagged FBW7 or both followed by either DMSO (-) or MG132 (10 μM) treatment for overnight. GFP plasmids were transfected as the normalization control for equal transfection efficiency. **C**. 293T cells were transfected with HA-tagged EglN2 in the presence of dominant negative FLAG tagged Cul1, 2, 3, 4a, 4b, 5 or control (-). Cell lysates were harvested followed by immunoblots as indicated. **D**. HCT-116 WT or *FBW7^−/−^* cells were treated with either control (-) or MG132 (10 μM) followed by immunoblots as indicated. Dotted vertical lane for EglN2 indicates that there is a non-essential lane that have been removed from a single original gel.

Previous research showed that EglN2 is an ER target gene that plays an important role in ER+ breast cancer [6, 7]. It remains unclear how EglN2 protein levels may be regulated in ER-, especially in TNBC. To further examine this, we examined EglN2 protein levels across a panel of established breast cancer cell lines. We divided these cell lines according to ER expression status (ER+ or ER-). Interestingly, a subset of ER- breast cancer cell lines (MCF-12A, MDA-MB-468, BT-549, Hs-578T, HCC1143 and MDA-MB-231) expressed substantial amount of EglN2, albeit slightly lower level than ER+ breast cancer cell lines including T47D, BT474 and ZR-75-1 (Figure 2A). Previous research showed that this subset of ER- breast cancer cell lines displayed *FBW7* loss [19]. Since FBW7 is an F-box protein that promotes substrate recognition by E3 ligase complex followed by the ubiquitination and degradation [22], it is reasonable to postulate that EglN2 may act as a potential substrate of the FBW7 E3 ligase complex in breast cancer. To test this hypothesis, we transfected EglN2 in the absence or presence of FBW7 and found that FBW7 expression decreased EglN2 protein levels in the cells, a process that can be rescued by concurrent treatment with the proteasomal inhibitor MG132 (Figure 2B). Consistently, the other two previously reported FBW7 substrates c-Myc and c-Jun [23] was downregulated by FBW7 expression, the effect rescued by MG132 treatment (Figure 2B). In contrast, the protein abundance of another EglN family member, EglN1, was minimally affected by FBW7 expression, suggesting the specific regulation of EglN2 by FBW7.

Since FBW7 is a Cullin1 (Cul1) based E3 ligase complex component, we hypothesize that inhibition of Cullin-based E3 ligases may also lead to EglN2 protein stability. To test that, we transfected cells with EglN2 in the presence of dominant negative Cul1, 2, 3, 4a, 4b and 5 mutants respectively as described previously [24]. These dominant negative mutants inhibited their respective E3 ligase complex activity. Consistently, dominant negative Cul1 led to an accumulation of EglN2 protein in cells, the effect recapitulated by MG132 treatment (Figure 2C). Notably, other known FBW7 substrates Cyclin E1, cMyc and cJun were similarly regulated by dominant negative Cul1 (Figure 2C). Interestingly, dominant Cul4b also led to EglN2 protein upregulation (Figure 2C). However, the nature of this finding remains to be determined with additional in-depth studies. To show that the proteasomal degradation dependent regulation of EglN2 by FBW7 may apply for other physiologically relevant system, as a complementary approach, we obtained HCT116 colon cancer cell lines with or without somatic *FBW7* knockout and found that EglN2 protein levels was upregulated by MG132 treatment in the cell line contain wild type FBW7, the effect mimicked by *FBW7* loss, suggesting that potential EglN2 regulation by FBW7 is mediated by proteasomal degradation pathway (Figure 2D). Since the *FBW7* genetic locus is in a region that is frequently deleted in TNBC [20] and the previous research showed that a significant portion of ER- breast cancer cell lines displayed *FBW7* loss [19], hereafter we particularly focus on the effect of FBW7 on EglN2 protein levels in breast cancer cell lines.

### Depletion of *FBW7* leads to increased EglN2 protein levels in breast cancer

**Figure 3:**
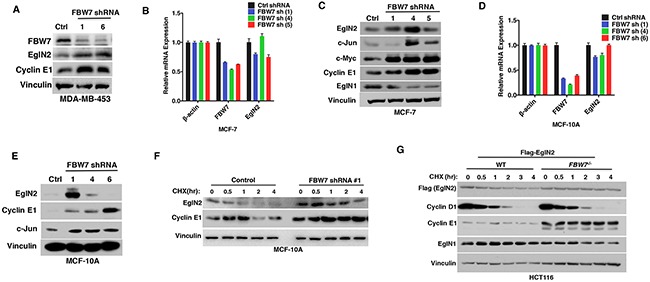
Depletion of FBW7 Leads to Increased EglN2 Protein Levels in Breast Cancer **A**. Immunoblot analysis of MDA-MB-453 cells infected with the lentivirus encoding FBW7 shRNA (#1, #6) or control (Ctrl) shRNA. **B-C**. qRT-PCR (B) or immunoblot (C) or analysis of MCF-7 cells infected with the lentivirus encoding either FBW7 shRNA (#1, #4, #5) or control (Ctrl) shRNA. **D-E**. qRT-PCR (D) or immunoblot (E) analysis of MCF-10A cells infected with the lentivirus encoding FBW7 shRNA (#1, #4, #6) or control (Ctrl) shRNA. **F-G**. Immunoblot analysis from cycloheximide (CHX) pulse chase of MCF-10A cells infected with the lentivirus encoding FBW7 shRNA (#1) or control (Ctrl) shRNA (F) or HCT-116 *WT* or *FBW7^−/−^* cells expressing exogenous Flag tagged EglN2 (G).

In order to examine the effect of FBW7 on EglN2 protein levels in breast cancer cell lines, we downregulated FBW7 expression levels by two independent hairpins in breast cell line MDA-MB-453 with wild type FBW7 expression and found FBW7 depletion led to increased EglN2 and Cyclin E1 levels (Figure 3A). Similarly, we depleted FBW7 expression by several independent hairpins in two other breast cell lines with functionally intact FBW7 (MCF-7 and MCF-10A). Real-time PCR confirmed the efficient downregulation of *FBW7* in these cell lines (Figure 3B and 3D). In addition, we also examined protein levels of other FBW7 substrates (Cyclin E1, c-Jun or c-Myc) in these cell lines to make sure of functional FBW7 downregulation. In most cases, we observed an increase in EglN2 protein abundance in *FBW7* depleted cell lines (Figure 3C and 3E), suggesting that FBW7 negatively regulated EglN2 protein levels in breast cancer cells. It is important to note that although qRT-PCR analyses showed similar FBW7 knockdown efficiency with different hairpins, the effects of these hairpins on protein levels of EglN2 or canonical FBW7 substrates (Cyclin E1, c-Jun or c-Myc) varied to some extent. This could reflect the possibility of some uncharacterized off-target effects for a particular FBW7 hairpin in a specific cell line. Notwithstanding this potential caveat, EglN2 protein upregulation by independent FBW7 hairpins in several different breast cancer cell lines (Figure 3) or somatic FBW7 knockout in HCT116 cells (Figure 2D) validates its negative regulation by FBW7. EglN2 mRNA levels were not robustly affected by any of FBW7 shRNAs, suggesting a potential post-transcriptional regulation of EglN2 by FBW7 (Figure 3B and 3D). To examine whether FBW7 depletion increased EglN2 protein stability in these cells, we treated MCF-10A cells with cycloheximide (CHX), an inhibitor for protein synthesis, followed by pulse chase with various time points. Depletion of FBW7 increased EglN2 protein stability in these cells (Figure 3F). Consistently, for HCT116 cells, somatic *FBW7* knockout increased exogenous EglN2 protein stability compared to wild type controls (Figure 3G). In summary, our data suggests that FBW7 depletion increases EglN2 protein stability.

### EglN2 C-terminal residues might mediate its negative regulation by FBW7

**Figure 4:**
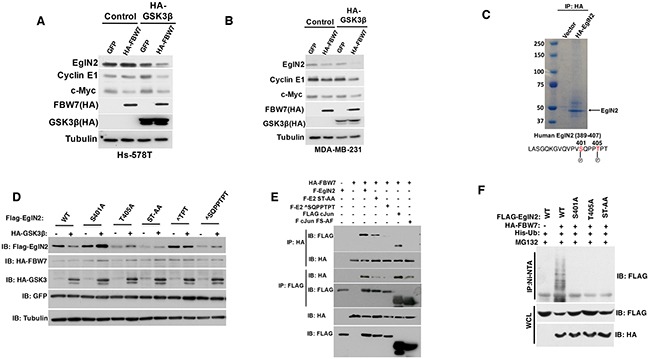
EglN2 C-terminal Residues Might Mediate Its Negative Regulation by FBW7 **A-B**. Immunoblot analysis of Hs-578T (A) and MDA-MB-231 (B) cells co-transfected with HA tagged FBW7 or GFP with either HA-tagged GSK3β or Control. Cell lysates were harvested 48 hours post-transfection. **C**. 293T cells were transfected with either HA tagged EglN2 or control followed by immunoprecipitation with HA agarose beads (3F10, Roche). Coomassie staining was performed to visualize the purified HA EglN2, which was cut followed by mass spectrometry analysis as described previously. EglN2 Ser401 and Thr405 sites were shown to be phosphorylated. **D**. Immunoblots for 293T cells co-transfected with various FLAG tagged EglN2 constructs (WT, S401A, T405A, ST-AA, ∧TPT or ∧SQPPTPT) and HA tagged GSK3β or Control (-). Equal amount of GFP was transfected to make sure of comparable transfection efficiency. Cell lysates were harvested 48 hours post-transfection. **E**. Immunoblot (IB) assays of whole cell extract (WCE) and immunoprecipitation (IP) of 293T cells (expressing Ctrl or HA-FBW7) co-transfected with Flag-EglN2 (F-EglN2), Flag-EglN2 ST-AA, Flag-EglN2 ∧SQPPTPT, Flag c-Jun or Flag c-Jun FS-AF mutants. Cells were treated with MG132 (10 μM) for overnight before harvesting at 48 hours post-transfection. **F**. *In vivo* ubiquitination assays of immunoprecipitation (IP with Ni-NTA) of 293T cells (expressing Ctrl or HA-FBW7) co-transfected with Flag-EglN2 (WT), Flag-EglN2 S401A, T405A or ST-AA. Cells were treated with MG132 (10 μM) for overnight before harvesting at 48 hours post-transfection.

The canonical FBW7 substrate such as Cyclin E1 or c-Jun is phosphorylated on threonine residues by GSK-3β followed by FBW7 E3 ligase complex recognition, ubiquitination and degradation [23]. We hypothesize that EglN2 may undergo GSK-3β dependent phosphorylation followed by FBW7 recognition and subsequent degradation. To test this hypothesis, we used two independent breast cancer cell lines that carry *FBW7* loss (Hs-578T and MDA-MB-231) and restored FBW7 protein function by using exogenous tagged FBW7. In the presence of exogenously tagged GSK-3β, there was a robust downregulation of EglN2 protein levels by FBW7 (Figure 4A-4B), indicating that GSK-3β-dependent phosphorylation may mediate EglN2 degradation by FBW7 recognition. To test this potential regulation of EglN2 by GSK-3β further, we searched the potential kinase site for human EglN2 sequence by using the previously established method ( scansite.mit.edu) and found that EglN2 Thr405 is a potential GSK-3β site, which is conserved across different mammalian species (data not shown).

Next, we purified HA tagged EglN2 in cells followed by examining its potential phosphorylate sites by mass spectrometry. Consistently, we observed that Thr405 and adjacent Ser401 sites were phosphorylated (Figure 4C), which are towards the very c-terminal of EglN2 coding sequence and the stop codon. In order to examine the potential role of these sites on regulating EglN2 protein levels in the cells, we generated a series of EglN2 mutants that contain the single or double mutations on these serines or threonines (S401A, T405A, ST-AA). Additionally, we also made the EglN2 deletion mutants that contain these serines or threonines (∧TPT or ∧SQPPTPT). Since these are very small truncation mutants that are next to the stop codon, we postulate that they may not affect the structural conformation of EglN2. Next, we co-transfected EglN2 WT or mutants with tagged GSK-3β and FBW7. It is important to note that the basal levels of WT or EglN2 mutants in the absence of GSK-3β displayed some variance, which awaits further investigation. Whereas wild type EglN2 was degraded in the presence of GSK-3β and FBW7, all of mutants escaped the GSK-3β and FBW7 mediated degradation (Figure 4D), strengthening the notion that EglN2 C-terminal Threonine or Serine residue may mediate its regulation by FBW7, potentially through GSK-3β mediated phosphorylation. Consistently, we observed the interaction between FBW7 and EglN2 in cells, whereas the binding diminished for EglN2 C-terminal mutants (Figure 4E), suggesting that EglN2 C-terminal may be important for its interaction with FBW7 therefore affecting its protein stability. Lastly, we examined the ubiquitination of EglN2 by FBW7 and found that FBW7 robustly promoted wild type EglN2 ubiquitination, the effect totally abrogated in EglN2 mutants that contain S401A, T405A or ST-AA mutations (Figure 4F) Accumulatively, our data suggests that EglN2 C-terminal sequence may mediate EglN2 binding with FBW7 followed by its ubiquitination, therefore contributing to its protein stability regulation.

## DISCUSSION

In this study, we showed for the first time that EglN2 plays an important role contributing to TNBC tumor growth. Mechanistically, *FBW7* loss in TNBC cells leads to upregulation of EglN2 protein levels. Hence, EglN2 protein stability is negatively regulated by the FBW7 E3 ligase complex. Depletion of *FBW7* in breast cancer cells results in EglN2 protein upregulation. In addition, we also identified several EglN2 phosphorylation sites on its C-terminal that may mediate its binding, ubiquitination and protein stability regulation by FBW7. Our findings suggest that EglN2 may contribute to TNBC tumor growth as a downstream target of FBW7.

There are multiple layers of protein regulation in the cells, including both transcriptional and post-transcriptional regulations. For example, in ER+ breast cancer, EglN2 is mainly regulated by estrogen and acts as a canonical estrogen inducible gene [6, 7]. In this setting, EglN2 contributes to Cyclin D1 regulation in an enzymatic dependent manner and regulates mitochondrial function in an enzymatic independent manner [8, 25]. From this perspective, it may be necessary to deplete *EglN2* protein level completely in order to have the durable anti-tumor efficacy *in vivo*. In ER- breast cancer, we report here that EglN2 protein stability is potentially regulated by SCF^FBW7^ E3 ligase complex. Interestingly, *FBW7* locates in a region that harbors the genomic loss frequently observed in TNBC [20]. TNBC patients display significant lower *FBW7* expression compared to normal breast controls and *FBW7* copy number loss predicts a worse prognosis (Zhang Q, unpublished data), indicating the important role of FBW7 in TNBC. Therefore, frequent loss of *FBW7* in TNBC may contribute to higher EglN2 protein levels and promote breast tumorigenesis. It remains to be determined whether the phenotype induced by FBW7 loss in TNBC is mediated through EglN2 or potentially other substrates. It will be also interesting to see how EglN2 contributes to TNBC and whether its enzymatic activity may be important for its effect on TNBC tumor progression.

Our findings further show that C-terminal of EglN2 may be important for its binding with FBW7, therefore contributing to its protein stability regulation by this E3 ligase complex. Consistently, we show that various EglN2 C-terminal mutants escape the degradation by FBW7. By mass spectrometry, we also identify two phosphorylation sites on EglN2 C-terminal Threonine (aa 405) and Serine (aa 401) residues. Interestingly, the Thr405 site is predicted to be a canonical GSK3β kinase site. Since GSK3β is reported to be the main kinase that promotes FBW7 substrate phosphorylation followed by the E3 ligase complex recognition [23], it is reasonable to hypothesize that Thr405 site may be a GSK3β kinase site that mediate EglN2 protein stability regulation by FBW7. Although our data show that FBW7 promotes EglN2 degradation in a GSK3β kinase dependent manner, we have not observed the GSK3β kinase induced EglN2 Thr405 site phosphorylation when performing the *in vitro* kinase assay with GST-tagged EglN2. There are several possibilities: First, it may be possible that EglN2 needs to be modified or primed in nearby residues in order for the Thr405 site to be efficiently phosphorylated by the GSK3β kinase. This is highly likely since we also observed that Ser401 site is phosphorylated *in vivo* by mass spectrometry. Second, it is also possible that there may be other unknown or non-canonical GSK3β kinase site in EglN2 sequences that need to be further characterized. Third, it is also likely that there may exist other kinases that may regulate EglN2 protein stability. In fact, by treating cells expressing exogenous EglN2 with different kinase inhibitors, we found that in addition to the GSK3β kinase inhibitor, the pan CDK inhibitor can also increase exogenous tagged EglN2 expression (Zhang Q, unpublished data).

Taken together, our results identify EglN2 as a potential therapeutic target in TNBC, and show that FBW7 may regulate EglN2 protein stability in breast cancer. *FBW7* loss induced EglN2 protein stabilization contributes to TNBC tumor progression, which offers a potential novel therapeutic avenue in treating this lethal disease.

## MATERIALS AND METHODS

### Cell cultures

293T, MDA-MB-157, MDA-MB-468, MDA-MB-453, HS-578T, MDA-MB-231, HCT116 cells were cultured in DMEM containing 10% fetal bovine serum (FBS) supplemented with 1% Pen/Strep. T47D, BT-474, ZR-75-1, HCC70, HCC1937, BT-549 and HCC1143 were cultured in RPMI medium containing 10% FBS and 1% Pen/Strep. MCF-10A and MCF-12A cells were cultured in DMEM/F12 media with 5% Horse serum, 20 ng/ml EGF, 100 ng/ml cholera toxin, 0.01 mg/ml insulin and 500 ng/ml hydrocortisone as described previously [26]. SKBR3 cells were maintained in McCoy5A media supplemented with 10% FBS +1% Pen/Strep. HCT116 WT and *FBW7*^−/−^ cells were kindly provided by Dr. Bert Vogelstein (Johns Hopkins University) [27] and were cultured in DMEM with 10% FBS +1% Pen/Strep as described previously [28]. Following lentivirus infections, cells were maintained in the presence of puromycin (2 μg/ml). All cells above were maintained at 37°C in 5% CO_2_ incubator.

### Western blot analysis and antibodies

Whole cell lysates were harvested by using EBC buffer (50 mM Tris pH8.0, 120 mM NaCl, 0.5% NP40, 0.1 mM EDTA and 10% Glycerol) supplemented with complete protease and phosphatase inhibitors (Roche Applied Biosciences). Cell lysate concentrations were measured by Bradford assay (Biorad) followed by SDS-PAGE analysis with equal amount of lysates supplemented with 3xSDS loading dye. Rabbit EglN2 antibody (NB100-310) was from Novus Biological. Rabbit anti Cyclin D1 (RB-010-P1) was from Neomarker. Antibodies against Vinculin (V9131), Tubulin (T9026) and FLAG (A8592) were from Sigma. Cyclin E1 (SC-247), c-Myc (SC-40), GFP (SC-9996) antibodies were from Santa Cruz. c-Jun (9165), p27 (3688) and EglN1 (3293S) antibodies were from Cell Signaling. HIF1α antibody (610959) was obtained from BD Bioscience. Mouse antibody against hemagglutinin (HA, MMS-101P) was obtained from Covance. Mouse Peroxidase conjugated goat anti-mouse secondary antibody (31430) and peroxidase conjugated goat anti-rabbit secondary antibody (31460) were purchased from Thermo Scientific.

### Immunoprecipitation

Cells were lysed in EBC lysis buffer supplemented with complete protease and phosphatase inhibitors (Roche Applied Bioscience). The lysates were cleared by centrifugation and then mixed with 3F10 HA conjugated beads (Roche Applied Bioscience) or FLAG M2 beads (Sigma) overnight. The bound complexes were washed with NETN buffer (120 mM NaCl, 20 mM Tris-HCl (pH 8.0), 0.5 mM EDTA, 0.5 % (v/v) Nonidet P-40 (NP-40) for 8 times and were eluted by boiling in SDS loading buffer. Bound proteins were resolved in SDS-PAGE followed by western blot analysis.

### Virus production and infection

293FT packaging cell lines was used for lentiviral amplification for EglN2 shRNAs or FBW7 shRNAs. Lentiviral infection was carried out similarly as previously described [8]. Briefly viruses were collected twice at 48 and 72 hours post-transfection with lipofectamine 2000 and helper plasmid pSPAX2 and pMD2G. After getting rid of the cell debris by passing through 0.45 μM filters, viruses were used to infect target cells in the presence of 8 μg/ml polybrene. Subsequently, target cell lines underwent puromycin (2 μg/ml) selection. Lentiviral shRNAs were obtained from Broad Institute TRC shRNA library. Target sequences are listed as follows:

Ctrl shRNA: AACAGTCGCGTTTGCGACTGG

EglN2 sh744: CGTTGAGTGTAGAGCTGAGAA

EglN2 sh746: GAATCAGAACTGGGATGTTAA

FBW7 shRNA #1: AACCTTCTCTGGAGAGA GAAA

FBW7 shRNA #4: CCAGAGAAATTGCTTGCTTTA

FBW7 shRNA #5: CCAGTCGTTAACAAGTGGAAT

FBW7 shRNA #6: CCAGAGACTGAAACCTGTCTA

### Plasmids

Dominant negative FLAG Cul1, 2, 3, 4a, 4b and 5 plasmids were obtained from Addgene. Flag-EglN2 was described in the previous publication [25]. HA tagged EglN2 was amplified by PCR with a 5’ primer that introduced a HA tag with a HINDIII site and a 3’ primer that introduced an EcoRI site. The PCR product was digested with HINDIII and EcoRI and cloned into the pcDNA3.1 vector cut with these two enzymes. HA tagged FBW7, HA-GSK3β, FLAG c-Jun and FLAG c-Jun FS-AF plasmids were described previously [29]. FLAG-EglN2 S401A, T405A, ST-AA, ∧TPT and ∧SQPPTPT were created by using site-directed mutagenesis (Agilent Technology). All plasmids were sequenced to make sure of correct sequence.

### Real-time RT-PCR

Total RNA was isolated with RNeasy mini kit (Qiagen). First strand cDNA was generated with the iScript cDNA synthesis kit (Biorad). Real time PCR was performed in triplicate as described before [8]. Real-Time RT-PCR primers used in this study are listed as below: β-actin (human) (Forward: AGAAAATCTGGCACCACACC; Reverse: GGGGT GTTGAAGGTCTCAAA) ; FBW7 (human) (Forward: TGGACCATGGTTCTGAGGTCCGC; Reverse: TTCGG CGTCGTTGTTGCCCT); EglN2 (human) (Forward: AACATCGAGCCACTCTTTGAC; Reverse: TCCTTG GCATCAAAATACCAG); Actin (mouse) (Forward: ACCAACTGGGACGACATGGA; Reverse: GGTCTCAA ACATGATCTGGGTCAT); EglN2 (mouse) (Forward: CTGGGCAACTACGTCATCAAT; Reverse: TGCACC TTAACATCCCAGTTC).

### Orthotopic tumor growth and animal experiments

Ten to eleven week old female FVB/NJ mice were used for xenograft studies. Approximately 4 x10^6^ viable C3Tag cells with either Ctrl shRNA or EglN2 shRNA (sh744) were resuspended in 100 μl growth factor reduced matrigel (BD biosciences) and injected orthotopically into the mammary gland as described previously [8]. Mice were sacrificed ten weeks after tumor implantation. The total mass of tumors was presented as mean ± SEM and evaluated statistically using a t test. *EglN2*^−/−^ mice were obtained from Regeneron Pharmaceuticals and were backcrossed to pure FVB/NJ background. C3Tag mice were obtained from Jackson Lab. For mice crossing between C3Tag and *EglN2*^−/−^, they were regularly monitored and euthanized when their tumor size reaches 20 mm (2.0 cm) in diameter. The survival time was calculated from the birth of mice to the day of sacrifice. All animal experiments complied with National Institutes of Health guidelines and were approved by the University of North Carolina at Chapel Hill Animal Care and Use Committee.

### Soft agar assay

First, 3% agarose (Sigma, A-4018) is prepared in water followed by the autoclave. Then, the agarose will be diluted with complete growth media to make 1% of agarose/media stock for the bottom layer. Then, 0.8% agarose will be made in order to mix with cells to make 0.4% agarose for the top layer. Cells were grown at 37°C in 5% CO_2_ incubator for three weeks followed by the staining with iodonitrotetrazolium chloride (Sigma) solution for staining and foci counting.
